# Solving Generalized Polyomino Puzzles Using the Ising Model

**DOI:** 10.3390/e24030354

**Published:** 2022-02-28

**Authors:** Kazuki Takabatake, Keisuke Yanagisawa, Yutaka Akiyama

**Affiliations:** Department of Computer Science, School of Computing, Tokyo Institute of Technology, Meguro-ku 152-8550, Tokyo, Japan; takabatake@bi.c.titech.ac.jp (K.T.); yanagisawa@c.titech.ac.jp (K.Y.)

**Keywords:** Ising model, polyomino puzzle, Hopfield neural network, combinatorial optimization

## Abstract

In the polyomino puzzle, the aim is to fill a finite space using several polyomino pieces with no overlaps or blanks. Because it is an NP-complete combinatorial optimization problem, various probabilistic and approximated approaches have been applied to find solutions. Several previous studies embedded the polyomino puzzle in a QUBO problem, where the original objective function and constraints are transformed into the Hamiltonian function of the simulated Ising model. A solution to the puzzle is obtained by searching for a ground state of Hamiltonian by simulating the dynamics of the multiple-spin system. However, previous methods could solve only tiny polyomino puzzles considering a few combinations because their Hamiltonian designs were not efficient. We propose an improved Hamiltonian design that introduces new constraints and guiding terms to weakly encourage favorable spins and pairs in the early stages of computation. The proposed model solves the pentomino puzzle represented by approximately 2000 spins with >90% probability. Additionally, we extended the method to a generalized problem where each polyomino piece could be used zero or more times and solved it with approximately 100% probability. The proposed method also appeared to be effective for the 3D polycube puzzle, which is similar to applications in fragment-based drug discovery.

## 1. Introduction

A polyomino, introduced by Golomb in 1954 [1], is a polygon formed by joining one or more squares edge to edge. Golomb studied the tiling problem using polyominoes, in which the goal of the problem is to cover an infinite or finite plane with replicas of a set of polyominoes [2,3,4]. The “polyomino puzzle” is a finite version of the tiling problem in which the goal is to cover a finite space using a number of replicas of a set of polyominoes with no overlaps or blanks. One of the most well-known polyomino puzzles is the pentomino puzzle, which is composed of twelve different pentomino pieces (formed by joining five squares) covering a 6×10 rectangular space, where each piece should be used only once.

The polyomino puzzle is known to be an NP-complete combinatorial optimization problem, and there is no polynomial-time exact algorithm to find its optimal solution. Therefore, various probabilistic and approximated algorithms have been proposed. Although heuristic tree search algorithms are conventionally used in such scenarios in computer science, they have difficulty finding optimal solutions with the desired probability as the scale of the problem increases.

In previous studies [5,6,7,8], the polyomino puzzle has been embedded in a quadratic unconstrained binary optimization (QUBO) problem and solved by simulating the dynamics of a multiple-spin system model inspired by the Ising model [9] in physics. The QUBO problem is a mathematical problem for which the objective is to find a vector of binary variables that minimizes the target quadratic polynomial function without any constraints on the variables. It is closely related to the Ising model with the quadratic Hamiltonian function, which has been thoroughly studied in statistical mechanics for over a century.

In the QUBO representation of the polyomino puzzle, each possible spatial placement of a specific polyomino is represented by a dedicated binary (or continuous) variable called a “spin”. Further, any mutually enhancing or repressing relationship between a pair of candidate spatial placements is represented by a connection coefficient between the corresponding spins. The solution to the puzzle is then obtained by searching for a ground state of the Hamiltonian function of the multiple-spin system. This is achieved by updating the system status based on the specific dynamics equations, which guarantees the local minimization of the Hamiltonian in time.

For the QUBO representation of the polyomino puzzle, the design of the Hamiltonian function and the dynamics model have been studied over more than three decades, including by Akiyama et al. [5], Takefuji et al. [6], and Manabe et al. [7]. Recently, the QUBO approach has been gaining increased attention because of the successes of quantum annealing, which has been applied to find global minimum solutions effectively. Eagle et al. [8] solved polyomino puzzles using a quantum annealing simulator and actual hardware DW2000Q (D-Wave Systems Inc., Burnaby, BC, Canada).

In the QUBO approach, the standard pentomino puzzle requires approximately 2000 spins at a minimum to correctly represent all possible placements of pieces, considering their rotation and inversion. However, the models used in previous studies could only deal with lower complexity problems than the pentomino puzzle, such as covering a small rectangular space and limiting the rotation and inversion of pieces. This is because their Hamiltonian designs were not efficient enough and overlooked factors such as the generation of unacceptable small blanks (bubble) where no other polyominoes can be placed. The quality of a Hamiltonian design strongly influences the possibility of reaching a global minimum with the QUBO approach whether using a digital computer or a quantum annealer. Therefore, the development of an improved Hamiltonian design strategy for such an application is necessary.

In this study, we propose a Hamiltonian of the QUBO model that effectively represents the polyomino puzzle with consideration of the bubbles and the introduction of novel guiding terms that weakly enhance favorable spins and pairs of spins in the early stages of calculation. We used the Hopfield neural network model [10,11,12] and evaluated the performance of the proposed Hamiltonian formulation for the standard pentomino puzzle, which is difficult to solve with previous methods (Section 3).

Furthermore, we expanded our method to the generalized version of the polyomino puzzle, in which each polyomino piece can be used zero or more times (not exactly once), allowing the target space to have an arbitrary shape (Section 4), and also accepting mixed polyomino sizes: tromino, tetromino, pentomino, hexomino, etc. (Section 5). These generalization efforts are important to enhance the applicability of this technique and pave the way for future real-world applications such as rapid virtual screening in fragment-based drug discovery, which can be regarded as a complicated version of the generalized polyomino puzzle proposed in this study.

## 2. Combinatorial Optimization Problem and the Ising Model

### 2.1. The Ising Model and the QUBO Problem

The Ising model—a simplified version of the Heisenberg model—is a mathematical model of ferromagnetism in statistical mechanics. In the Ising model, spins that can be in either of two states (+1 or −1) are typically located on the lattice points, and there are interactions between adjacent spins. The system has a quadratic Hamiltonian function and the spin status changes such that it minimizes the Hamiltonian spontaneously through the interaction between adjacent spins.

The Ising model is mathematically equivalent to the QUBO problem of finding a binary vector that minimizes the target quadratic polynomial function without any constraints on the variables. Lucas formulated various NP-hard combinatorial optimization problems, such as the maximum cut problem and the traveling salesperson problem, as Ising models [13]. He embedded the objective function and constraints of the combinatorial optimization problem into a corresponding QUBO problem by adding penalty terms in the Hamiltonian function that raise the energy of a state only when any of the required constraints are violated.

Nowadays, further efforts are underway to formulate real-world application problems as QUBO problems, such as financial portfolio optimization running on a machine from D-Wave [14] and drug discovery with a Gaussian boson sampling simulator [15]. Additionally, the quantum annealer, or the hardware architecture harnessed by quantum entanglement dedicated to solving the QUBO problems based on the Ising model, has been actively developed. Examples include the quantum annealing machine from D-Wave Systems [16] and the coherent Ising machine (CIM) from NTT [17]. Furthermore, software simulators running on general-purpose computers such as the simulated bifurcation machine (SBM) from Toshiba [18] and dedicated but classical semiconductor chips such as the Digital Annealer from Fujitsu [19] have also been developed.

### 2.2. Hopfield Neural Network for Solving Combinatorial Optimization Problems

In the field of computer science, mathematical models inspired by the Ising model, such as the Hopfield neural network [10,11,12], have been studied as an exotic computing approach to solve QUBO problems. The Hopfield neural network, proposed by Hopfield in 1982 [10], is similar to the electron spin-glass model and can be used to solve QUBO problems by utilizing the intrinsic characteristics of its quadratic Hamiltonian minimization. In the original model, the output of a neuron, which is a node of the network, had a binary output value of zero or one. In the later model, the output has been extended to have a continuous value from zero to one [11]. Further, the spin status is computed as continuous values, and a binarization operation is performed after reading out the final status.

The Hopfield neural network is able to solve combinatorial optimization problems by embedding the objective function and constraints into a QUBO formulation and searching for a ground state of Hamiltonian defined for the network [12]. The procedure merely provides a local minimum of Hamiltonian depending on the initial states and random noise, and thus many trials are required in general to find the ground state. A carefully designed Hamiltonian function that efficiently represents the characteristics of the target problem and provides a more convex energy landscape can significantly increase the probability of reaching the global minimum. An appropriate procedure for updating the system status can also significantly influence the convergence performance for finding the optimal solution in the Hopfield neural network and its derivative algorithms, such as the Gaussian machine [20]. In this study, we evaluated the performance of the proposed method by using a software simulator that implements a Hopfield neural network with continuous output values.

Considering the Hopfield neural network, the energy ground state is searched for by iteratively updating the neurons’ output based on specific update rules. Regarding the model with continuous output values, each neuron first calculates its internal value $U_{i}$ using the following differential equation:

(1) $$\frac{dU_{i}}{dt}=-\frac{U_{i}}{\tau }+\sum_{j=1}^{n}W_{ij}V_{j}+\theta_{i}\;,$$

where n is the number of neurons in the network, $W_{ij}$ is the weight between neurons i and j, $V_{j}$ is the output of another neuron j, and $\theta_{i}$ is the bias (i.e., the input to neuron i). In addition, τ is the time constant parameter, which determines how well the internal values of the neuron are conserved, considering the time variation dt. The Hopfield neural network is an interconnected network; however, it does not contain any self-connections ($W_{ii}=0$), and the weight values are symmetric ($W_{ij}=W_{ji}$).

Thereafter, the output value $V_{i}$ of the neuron i is determined by the following sigmoid function:

(2) $$V_{i}=\frac{1}{1+\exp\left(-U_{i}/T_{mf}\right)}\;,$$

where $T_{mf}$ is a parameter that determines the gradient of the sigmoid function (also known as the mean-field temperature). A higher $T_{mf}$ makes the sigmoid function closer to a linear function, whereas a lower $T_{mf}$ makes it closer to a step function.

The energy of the network in the continuous value model is defined as follows:

(3) $$F=E-T_{mf}\cdot S\;,$$

(4) $$E=-\frac{1}{2}\sum_{i=1}^{n}\sum_{j=1}^{n}W_{ij}V_{i}V_{j}-\sum_{i=1}^{n}\theta_{i}V_{i}\;,$$

(5) $$S=\sum_{i=1}^{n}\left\{V_{i}\log_{e}\frac{1}{V_{i}}+\left(1-V_{i}\right)\log_{e}\frac{1}{1-V_{i}}\right\}\;.$$

The discrete value model minimizes the energy E in Equation (4) by repeatedly updating the neurons, whereas the continuous value model minimizes the energy F, which also considers the entropy term S as in Equation (3) [11]. Here, it is possible to search for a solution to a combinatorial optimization problem using the following steps.

1. Express the objective function to be minimized in a quadratic form for n binary variables.
2. Compare the quadratic form to be minimized with the energy function E in Equation (4) and obtain the values of $W_{ij}$ and $\theta_{i}$, which make them coincide.
3. Construct a Hopfield neural network with the obtained values of $W_{ij}$ and $\theta_{i}$.
4. Select one neuron randomly and update it using Equations (1) and (2).
5. Perform Step 4 several times to minimize the free energy F in Equation (3).
6. Obtain the output values of the neurons and binarize them based on some criteria. The solution is obtained if the output values of the neurons after the binarization operation satisfy the constraint conditions. In this study, the binarization is performed based on whether the output value is greater than or equal to 0.5.

Regarding this case, for the binarized result of the minimum solution to F to be consistent with the minimum solution to E, the value of $T_{mf}$ must be considerably small at the end. Moreover, there must be no significant difference between the landscape of the basin created by F and that of E. Akiyama et al. proposed a method known as the sharpening technique, in which $T_{mf}$ is large and the gradient of the sigmoid function is gradual in the initial states and becomes progressively steeper over time [5]. This method increases the probability of convergence to the global minimum by gradually transforming the space of F from a state where the ground state is easy to find to a state where there is almost no difference from the original landscape of E. We employ this technique, and the details of $T_{mf}$ scheduling are presented in Section 3.3.1.

## 3. Solving Pentomino Puzzles with the Ising Model

### 3.1. Pentomino Puzzle

**Figure 1:**
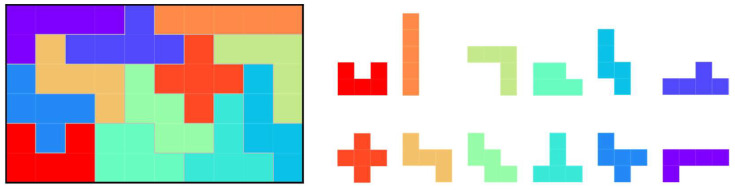
The 6×10 board and pentominoes used in the pentomino puzzle.

Figure 1 shows a pentomino puzzle, which is a well-known polyomino puzzle example. Pentominoes are formed by joining five squares, and each of the twelve unique pentominoes is used only once in the pentomino puzzle. Therefore, the total size of the board is 60 squares, and a typical example is a 6×10 board, as shown in Figure 1. In this study, pentominoes can rotate every 90 degrees and can be inverted (flipped front and back). Considering these conditions, the pentomino puzzle has 2339 unique solutions [21].

### 3.2. Improvement of the Hamiltonian Function

**Figure 2:**
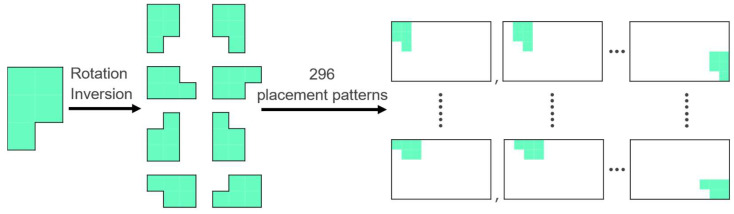
Correspondence between neurons in the Hopfield neural network and placement patterns.

Applying the Hopfield neural network to the pentomino puzzle, we referred to previous studies to design the neurons; specifically, a neuron corresponds to a single pattern of placement of each pentomino on the board (Figure 2) [5,22]. If the output of a neuron is one, it indicates that a pentomino is at the spatial corresponding position of the neuron. For instance, there are a total of 296 possible placement patterns on the board, considering the rotation and inversion of the specific pentomino piece shown in Figure 2. Similarly, considering the number of placement patterns for the other eleven types of pentominoes, we observe that a total of 1928 neurons are required to represent all the placement patterns of the 12 pentominoes.

Considering the design of the Hamiltonian function, which includes the biases applied to neurons and the connection weights between neurons, we improve the formulation by adding new terms, compared to previous studies [5,22]. Here, we first describe the two conventional constraint terms. Subsequently, we describe four newly proposed terms.

#### 3.2.1. Constraint on the Number of Times Each Pentomino Is Used

All pentominoes must be placed somewhere on the board only once. This constraint can be expressed by the following equation:

(6) $$H_{1}=\frac{1}{2}\overset{M}{\underset{m=1}{\sum }}{\left(\underset{i\in P_{m}}{\sum }V_{i}-1\right)}^{2}+\frac{1}{2}\overset{M}{\underset{m=1}{\sum }}\underset{i\in P_{m}}{\sum }V_{i}\left(1-V_{i}\right)\;,$$

where M is the number of types of pentominoes and $P_{m}$ is the set of neurons that express all the placement patterns of a pentomino m. The first term of Equation (6) becomes zero only when the summation of $V_{i}$ included in $P_{m}$ is one for each pentomino m. An ideal state in which the output of only a single neuron is one and others are zero for each set of neurons $P_{m}$ satisfies the condition. The second term is to eliminate $W_{ii}$ (the weight from a neuron back to itself) generated by the first term; therefore, $W_{ii}=0$.

#### 3.2.2. Constraint on the Overlap of the Pentominoes on the Board

All pentominoes must be placed with no overlaps or blanks as a solution to the pentomino puzzle. This constraint can be expressed by the following equation:

(7) $$H_{2}=\frac{1}{2}\overset{N}{\underset{n=1}{\sum }}{\left(\underset{i\in L_{n}}{\sum }V_{i}-1\right)}^{2}+\frac{1}{2}\overset{N}{\underset{n=1}{\sum }}\underset{i\in L_{n}}{\sum }V_{i}\left(1-V_{i}\right)\;,$$

where N is the number of squares constituting the board, and $L_{n}$ is the set of neurons that express all the placement patterns of the pentominoes occupying a square n on the board. The first term of Equation (7) becomes zero only when the summation of $V_{i}$, included in $L_{n}$, is one for each square n on the board. An ideal state where the output of only a single neuron is one and the others are zero for each set of neurons $L_{n}$ satisfies this condition. If multiple neurons in a set of neurons corresponding to a specific square have an output value of one, it indicates that pentominoes are overlapping on that square. Furthermore, all neurons in the set have an output value of zero, indicating that the square is left blank. The second term in the equation is to eliminate $W_{ii}$, which is the same as the second term of the $H_{1}$ described in Section 3.2.1.

#### 3.2.3. Inhibition of Bubbles

**Figure 3:**
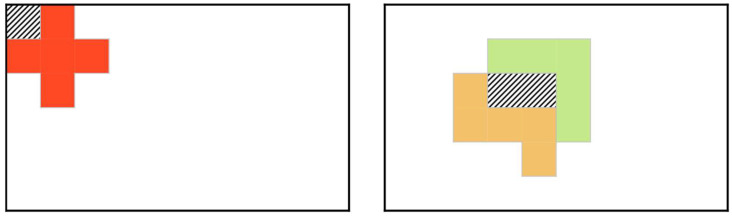
Bubbles generated by a single pentomino and a pair of pentominoes.

In addition to the two constraints presented above, we added a new constraint to inhibit bubbles, which are small blanks where there is no room for another pentomino to be inserted, as demonstrated in the shaded areas in Figure 3. Bubbles can be generated by a single pentomino or by a combination of several pentominoes. The placement of a single pentomino that generates a bubble cannot be included in any solution; thus, it can be easily excluded beforehand. In contrast, it is desirable to restrict a pair of neurons that generate bubbles; therefore, the following term is added to the Hamiltonian:

(8) $$H_{3}=\frac{1}{2}\overset{Q}{\underset{i=1}{\sum }}\overset{Q}{\underset{j=1}{\sum }}f\left(i,j\right)V_{i}V_{j}\;,\;f\left(i,j\right)=\{\begin{array}{rr} 1 & \mathrm{if}\;\mathrm{neurons}\;i,j\;\mathrm{generate}\;a\;\mathrm{bubble}\;,\\ 0 & \mathrm{otherwise}\;, \end{array}$$

where Q is the total number of neurons, and the function f(i,j) represents whether the bubbles are generated or not. If all the pairs in the set of neurons with an output value of one do not generate bubbles, then this term is minimized to zero.

#### 3.2.4. Encouragement for Combinations of Pentominoes That Contact Each Other

Considering the $H_{3}$ described in Section 3.2.3, we restrict inappropriate combinations of neurons that generate bubbles. In contrast, it is also possible to improve the convergence performance to the optimal solution by encouraging appropriate combinations of neurons. One of the examples is the appropriate mutual contact of pentominoes. The more the edges of two pentominoes touch, the better the contact between them. Therefore, the following term is added to the Hamiltonian:

(9) $$H_{4}=-\frac{1}{2}\overset{Q}{\underset{i=1}{\sum }}\overset{Q}{\underset{j=1}{\sum }}g\left(i,j\right)V_{i}V_{j}\;,\;g\left(i,j\right)=\{\begin{array}{rr} \mu_{ij} & \mathrm{if}\;\lambda_{ij}=0\;,\\ 0 & \mathrm{otherwise}\;, \end{array}$$

where $\mu_{ij}$ is the number of edges of squares shared by the two pentominoes corresponding to the neurons i,j, and $\lambda_{ij}$ is the number of overlapping squares of the two pentominoes. The function g(i,j) evaluates the goodness of the combination for all pairs of neurons. If the placements of pentominoes corresponding to neurons i,j do not overlap, then the pair is encouraged in proportion to the number of edges they share with each other.

#### 3.2.5. Encouragement for Pentominoes That Contact the Borders of the Board

Regarding the $H_{4}$ described in Section 3.2.4, we evaluate the goodness of the combinations of pairs of neurons. Similarly, we can also evaluate the goodness of a single neuron. To weakly encourage pentominoes that contact the borders of the board well, the following term is added to the Hamiltonian:

(10) $$H_{5}=-\overset{Q}{\underset{i=1}{\sum }}h_{i}V_{i}\;,$$

where $h_{i}$ is the number of edges where the placement of a pentomino corresponding to neuron i contacts any borderline of the board.

#### 3.2.6. Broad Restriction on All Pairs of Neurons

The encouragement terms described above will promote the finding of an optimal solution; however, this tends to make output values of several neurons fixed to one at the early stages in the simulation. It reduces the simulation runtime. Nonetheless, it increases the probability of being trapped in a local minimum because the Hopfield neural network is sensitive to the initial states of neurons initialized by uniform random numbers. To avoid this unintentional behavior, all pairs of neurons are equally suppressed. Thus, the following term is added to the Hamiltonian:

(11) $$H_{6}=\frac{1}{2}\overset{Q}{\underset{i=1}{\sum }}\overset{Q}{\underset{j=1}{\sum }}V_{i}V_{j}\;.$$

#### 3.2.7. Hamiltonian for the Pentomino Puzzle

Combining Equations (6)–(11), the modified Hamiltonian for the pentomino puzzle is proposed as follows:

(12) $$H=AH_{1}+BH_{2}+CH_{3}+\gamma \left(DH_{4}+EH_{5}+FH_{6}\right)\;.$$

Whereas $H_{1}$ to $H_{3}$ are constraint terms that describe the hard constraints on the problem, $H_{4}$ to $H_{6}$ are the guiding terms that work to guide the system to the global minimum. Guiding terms that encourage or restrict neurons or pairs of neurons have a common issue. If the coefficient of the term is too large, the ground state of the free energy function does not necessarily match the state in which the target puzzle is solved. Therefore, we introduce the parameter γ, which is a decaying weight coefficient for guiding terms. The parameter γ is one at the start of the simulation and decays linearly to zero at the end. Therefore, the effect of guiding terms is weakened over the course of the simulation. The coefficients A, B, C, D, E, and F on each term are positive real numbers. These coefficients determine the balance of each term; therefore, it is very important to select the appropriate values.

Comparing Equation (4) to Equation (12), the weights $W_{ij}$ between the neurons and biases $\theta_{i}$ are obtained as follows:

$$W_{ij}=\{\begin{array}{rr} -A-B\lambda_{ij}-Cf\left(i,j\right)+D\gamma g\left(i,j\right)-F\gamma & \mathrm{if}\;i\neq j\;\mathrm{and}\;i,j\in P_{m}\;,\\ -B\lambda_{ij}-Cf\left(i,j\right)+D\gamma g\left(i,j\right)-F\gamma & \mathrm{if}\;i\neq j\;\mathrm{and}\;i\in P_{m}\;\mathrm{and}\;j\notin P_{m}\;,\\ 0 & \mathrm{otherwise}\;, \end{array}\;\theta_{i}=\frac{A}{2}+\frac{B}{2}a_{i}+E\gamma h_{i}\;,$$

where $a_{i}$ is the size of the polyomino corresponding to neuron i ($a_{i}$ is five for the pentomino puzzle).

### 3.3. Computer Experiments

#### 3.3.1. Experimental Parameters

Considering the simulation, we randomly selected one of the n neurons and calculated its output value $V_{i}$ based on the update rule described in Section 2.2. In other words, we chose the asynchronous update of neurons, which is only feasible in software simulations and has a better effect for faster convergence. In this study, we define one time step as performing this update process n times, which is the number of neurons. Because the selection of a neuron was performed randomly, there were neurons that were updated multiple times and some that were never updated during a single step. Moreover, Δt=1 and τ=1 are used in Equation (1), which indicates that the influence of the previous value of $U_{i}$ is completely forgotten in each update. In addition, the output value $V_{i}$ of each neuron is initialized by a uniform random number in the range [0, 2/n] such that the sum of the outputs of the n neurons approaches one. This is because if each output value is too large, such as 0.5, it will take longer for the network to stabilize at the earlier stages of the simulation.

We employed the sharpening technique that decays $T_{mf}$, which determines the gradient of the sigmoid function over the course of the simulation. The scheduling of $T_{mf}$ is as follows:

$$T_{mf}\left(t\right)=\max\left(T_{mf}^{\left(0\right)}\left(1-\frac{t}{t_{max}}\right),10^{-6}\right)\;,$$

where $T_{mf}^{\left(0\right)}$ is the initial value of $T_{mf}$, which is fixed at one in this experiment. Moreover, t is the number of steps in the simulation, and $t_{max}$ is the total number of steps to be specified. The computer experiments presented in this section were performed by varying $t_{max}$ from 500 to 5000.

The coefficients of the proposed Hamiltonian were determined via Bayesian optimization to obtain the values shown in Table 1. The details are described in Section 6.1.

#### 3.3.2. Probability of Finding the Optimal Solution

**Figure 4:**
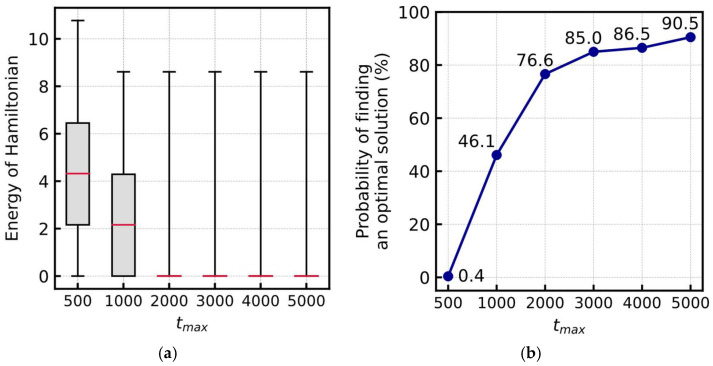
(**a**) Energy distribution of Hamiltonian and (**b**) probability of finding an optimal solution for various $t_{max}$.

Figure 4 shows the experimental results obtained by changing the total number of simulation steps, $t_{max}$. Considering each setting of $t_{max}$, we ran the simulation 1000 times with different random seeds for generating the initial output value of the neurons. Figure 4a shows the distribution of the Hamiltonian energy obtained in the final state of the simulation. As $t_{max}$ increases, the system discovers smaller energy states, indicating better solutions. Figure 4b shows the probability of finding the optimal solution, where the probability is 90.5% at $t_{max}=5000$, indicating that the proposed Hamiltonian has a better performance in searching for the solution.

The CPU time for a simulation with $t_{max}=5000$ using a single CPU core (Intel Xeon E5-2680 2.4 GHz, Intel Corporation, Santa Clara, CA, USA) was approximately 30 s. 

**Figure 5:**
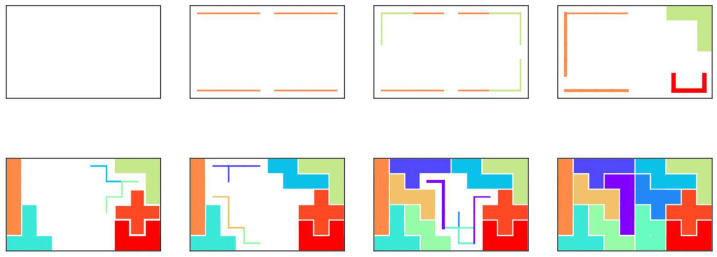
Series of intermediate states in the course of conversion to an optimal solution. Each pentomino placement corresponds to a neuron, and the thickness of each pentomino represents the output value of the neuron.

Figure 5 shows the intermediate states of conversion to an optimal solution. Considering any trial, the pentominoes in contact with the borders were placed first, mainly because of the $H_{5}$ term. Additionally, the $H_{2}$ term, which restricts the overlap of pentominoes, suppresses the placement to the center of the board where pentominoes are easily overlapped. Thereafter, the placement of pentominoes that have been in contact with the already placed pentominoes can be selected. This is because the $H_{4}$ term encourages good pairs of placements and when complex spaces are produced by the placement of pentominoes, only specific pentominoes can cover the space properly. Thus, because the proposed method is not formulated in such a manner as to increase the diversity of solutions, it tends to converge to the same optimal solution in multiple trials. When the number of steps $t_{max}=5000$, the optimal solution was found in 905 out of 1000 trials, with only 4 unique solutions.

## 4. Allowing Zero or Multiple Usage of Polyomino and Arbitrarily Shaped Board

### 4.1. Target Problem

The pentomino puzzle presented above can be considered a special case of the generalized problem. In this section, we extend the puzzle in two aspects as a first step:

1. Each pentomino can be used zero or more times;
2. An arbitrarily shaped board is the subject of the problem.

**Figure 6:**
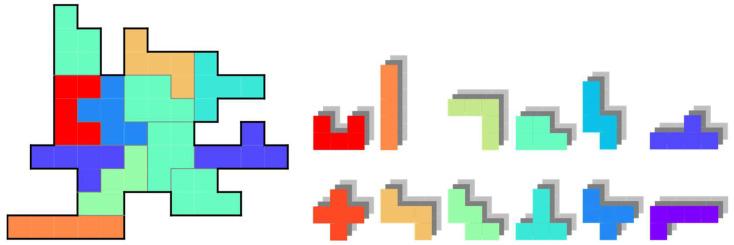
Example of a generalized polyomino puzzle with pentominoes.

Hereinafter, the generalized model allows each polyomino to be used zero or more times. Additionally, we consider boards of any shape (all the squares making up the board need to be interconnected) as a crucial step for approaching real-world applications. Figure 6 shows an example of the generalized polyomino puzzle problem.

### 4.2. Hamiltonian for the Generalized Polyomino Puzzle

For the generalized polyomino puzzle described in Section 4.1, a modification of the Hamiltonian in Equation (12), which is for pentomino puzzles, needs to be considered. Because none of the terms in Equation (12) depend on the shape of the target board, the same Hamiltonian can be used for the arbitrary shape of the board. In contrast, because the $H_{1}$ term restricts the number of times each pentomino can be used, this $H_{1}$ must be removed to place each pentomino zero or more times. Therefore, the Hamiltonian for the generalized polyomino puzzle is introduced as follows:

(13) $$H=BH_{2}+CH_{3}+\gamma \left(DH_{4}+EH_{5}+FH_{6}\right)\;.$$

The weights $W_{ij}$ between the neurons and biases $\theta_{i}$ are obtained as follows:

$$W_{ij}=\{\begin{array}{rr} -B\lambda_{ij}-Cf\left(i,j\right)+D\gamma g\left(i,j\right)-F\gamma & \mathrm{if}\;i\neq j\;,\\ 0 & \mathrm{otherwise}\;, \end{array}\;\theta_{i}=\frac{B}{2}a_{i}+E\gamma h_{i}\;.$$

### 4.3. Generation of Arbitrarily Shaped Boards

**Figure 7:**
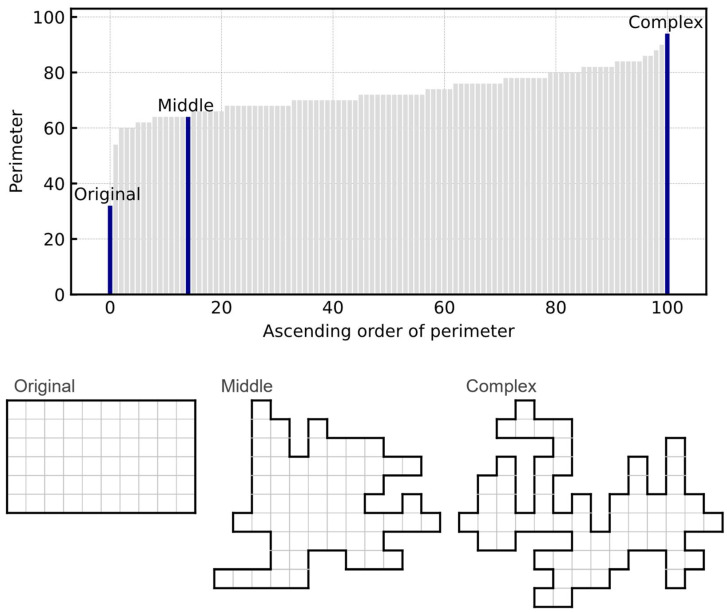
Complexities (perimeters) of the randomly generated boards, and the shape of the three selected boards.

We generated several boards with complex shapes for the generalized polyomino puzzle other than the 6×10 board used in the pentomino puzzle. Each board was randomly generated by joining 60 squares without any hole on the board. Figure 7 shows the 101 different boards in an ascending order based on their perimeters, representing their complexity. This consists of the original 6×10 board and 100 randomly generated boards. We selected the 6×10 board with a perimeter of 32 as the original board (Original), and the board with the longest perimeter of 94 was considered as the most complex board (Complex). Furthermore, the board with a perimeter of 64, which is closest to the average of those two perimeters, was chosen as the board with the average complexity (Middle). The total number of neurons for the Original, Middle, and Complex boards was 1928, 857, and 129, respectively. These three boards are shown in Figure 7.

### 4.4. Computer Experiments

#### 4.4.1. Experimental Parameters

The experiments were performed with the total number of steps $t_{max}$ changing from 50 to 500. The steps were set shorter than in the experiment described in Section 3.3.1 because the number of simulation steps required to obtain an optimal solution was smaller. We used Bayesian optimization to determine the coefficients of the proposed Hamiltonian function, shown in Table 2. For the other parameters, we used the same settings as in Section 3.3.1.

#### 4.4.2. Probability of Finding an Optimal Solution

**Figure 8:**
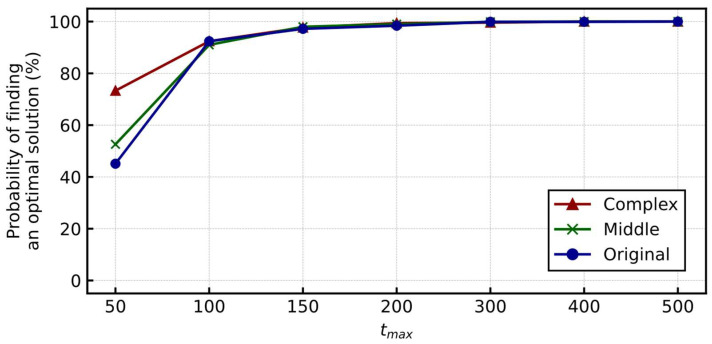
Probability of finding an optimal solution for the various $t_{max}$ (generalized polyomino puzzles).

Figure 8 shows the probability of finding an optimal solution for various $t_{max}$. Similar to Section 3.3.2, considering each $t_{max}$, we ran the simulation 1000 times with different random seeds for generating the initial output value of the neurons. More than 99% of the trials reached an optimal solution at $t_{max}=200$ for each board, indicating that the proposed Hamiltonian had a sufficient performance for these problems.

Moreover, the probability of the Original board was substantially higher than the standard pentomino puzzle shown in Section 3.3.2, even with fewer $t_{max}$. The extension of the number of times each pentomino is used can be considered as the drastic relaxation of the problem, resulting in easier achievement. In addition, the overall probability of finding an optimal solution in the Middle and Complex boards was higher than that in the Original. This is because of their complex shapes, where the total number of neurons is lower because there are areas with fewer candidates suitable to cover the space. Moreover, the output values of certain neurons are fixed to one in the early stages of the simulation, narrowing the search space.

**Figure 9:**
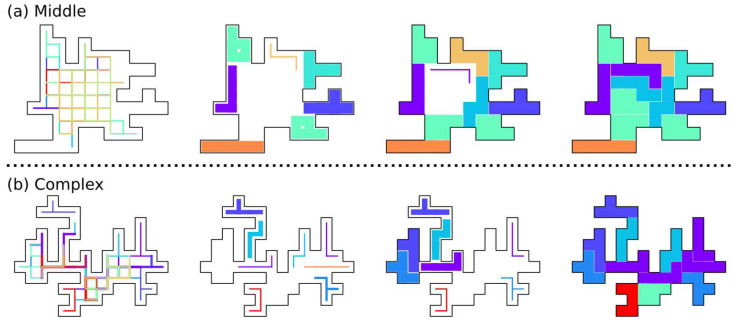
Series of intermediate states in the course of the conversion to an optimal solution for the (**a**) Middle and (**b**) Complex boards.

Figure 9 shows the intermediate states in the course of conversion to an optimal solution of Middle (Figure 9a) and Complex (Figure 9b) boards. Because of the complex shapes of the board, some places have only a few candidates for pentominoes that can cover them, and the output value of the corresponding neuron is fixed to one at the earlier stages of the simulation.

## 5. Accepting Various Sizes of Polyominoes

### 5.1. Set of Polyominoes

Popular polyomino puzzles use polyominoes of the same size. However, some problems deal with various sizes of polyominoes, such as the example of a commercialized polyomino puzzle, where an 8×8 board is covered with twelve pentominoes and one tetromino.

**Figure 10:**
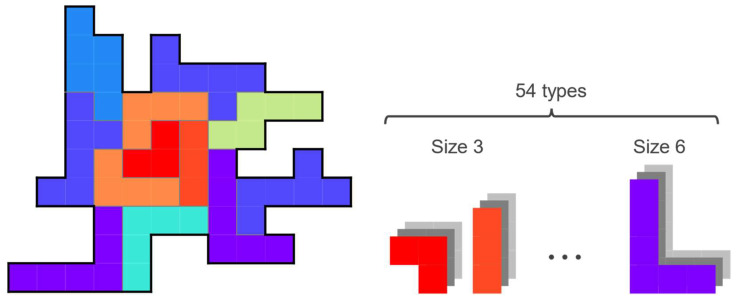
Example of the generalized polyomino puzzle with polyominoes of various sizes.

Figure 10 is an example of the problem discussed here. In addition to the extensions described in Section 4, we further extend the puzzle to deal with polyominoes of various sizes simultaneously as a subsequent step. However, covering a board with small polyominoes is generally easier than with large polyominoes. Regarding some industrial applications, such as fragment-based drug discovery, it is desirable to cover the space by combining a small number of larger and functional components, rather than simply filling it up with several tiny components. Therefore, we consider the problem of minimizing the total number of polyominoes used by enhancing the use of larger polyominoes.

Table 3 shows the number of types of polyominoes for each size. Polyominoes that have the same shape when rotated or inverted are treated as the same type of polyomino. In addition to the twelve types of pentominoes discussed in the previous sections, we introduced new polyominoes of various sizes. However, if we include tiny polyominoes such as the monomino (size 1) and the domino (size 2) in the candidate set, there are trivial solutions, such as covering the board by simply repeating them. Therefore, only the tromino (size 3) and the tetromino (size 4) were included as small-size polyominoes. We also included the hexomino (size 6) as larger polyominoes. We have not included heptomino (size 7) and those larger than that because they have a large number of shapes and the total number of neurons required will be too large. The total number of types of polyominoes used was 54. When all the 54 polyominoes were used, the total number of neurons needed for the Original, Middle, and Complex boards was 8469, 4141, and 693, respectively.

### 5.2. Computer Experiments

#### 5.2.1. Experimental Parameters

Considering the generalized polyomino puzzle with various sizes of polyominoes, we used the same Hamiltonian as in Section 4 because the $H_{6}$ term could reduce the total number of polyominoes to be used. A detailed discussion is presented in Section 5.2.3. The experiments were performed by varying the total number of steps $t_{max}$ from 50 to 500. We used Bayesian optimization to determine the coefficients of the proposed Hamiltonian, which are shown in Table 4. For the other parameters, we used the same settings as in Section 3.3.1.

#### 5.2.2. Probability of Finding an Optimal Solution

**Figure 11:**
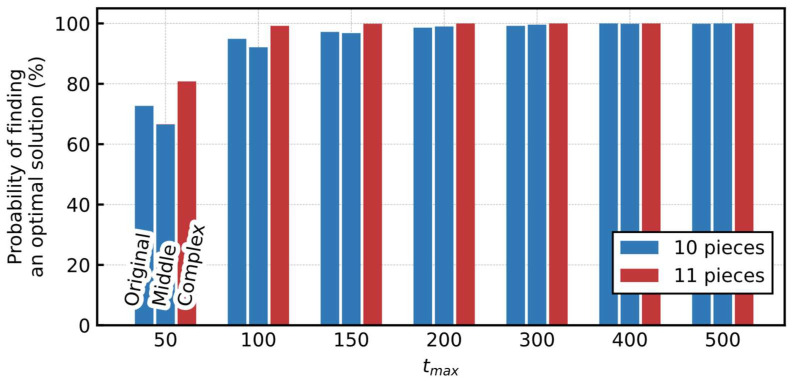
Probability of finding an optimal solution and the breakdown of the number of placed polyominoes of the optimal solution for various $t_{max}$.

Figure 11 shows the probability of finding an optimal solution and the breakdown of the total number of polyominoes used in each solution. Considering each $t_{max}$, we ran the simulation 1000 times with different random seeds to generate the initial output value of the neurons. There was a little difference in the probability of finding an optimal solution among the three boards, and the probabilities were reached at approximately 100% at $t_{max}=200$ or larger for all the boards. The probability of finding a solution does not differ significantly compared to the results using only the pentominoes shown in Figure 8. This is because although the total number of required neurons was increasing, the total number of possible solutions was also increasing simultaneously. In addition, considering the breakdown of the total number of polyominoes used in the obtained solutions, most of the solutions in the Original and Middle boards covered the 60 squares using only 10 hexominoes (polyominoes of size 6), minimizing the total number of polyominoes. There was no solution that covered the Complex board with ten polyominoes; therefore, the solutions with eleven polyominoes were obtained.

#### 5.2.3. Size of the Placed Polyominoes

**Figure 12:**
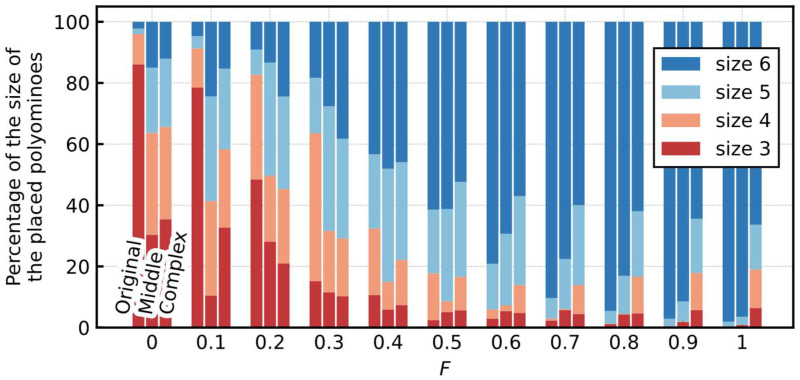
Breakdown of the size of the placed polyominoes for various F. It includes the results of all the trials, not only those in which an optimal solution is found. Moreover, $t_{max}$ of all the trials was fixed at 500, which was sufficiently large, considering the probability of finding an optimal solution.

In the previous section, we showed that the total number of polyominoes used to cover the board was minimized by using the same Hamiltonian as in Section 4.4.1, which was not an intuitive result because the effect of the $H_{6}$ term suppresses all the pairs of neurons universally. The $H_{6}$ term penalty increases when the number of neurons to output one is increasing, indicating the use of more polyominoes. Therefore, the use of fewer and larger polyominoes was encouraged to minimize the Hamiltonian energy, and the total number of polyominoes was minimized. Figure 12 shows the breakdown of the size of the placed polyominoes when the coefficient F for the $H_{6}$ term varies from the optimum value obtained shown in Table 4. At F=0, a large proportion of trominoes and tetrominoes are observed. However, as coefficient F increases, the proportion of such small-sized polyominoes decreases, and at the setting of F=1, hexominoes are mostly used. The same is true for F>1. For the Complex board, a certain percentage of tetrominoes and pentominoes were used, even at F=1 or larger, because the board contained areas that were not coverable only with hexominoes.

#### 5.2.4. Performance with the Reduced Types of Polyominoes

**Figure 13:**
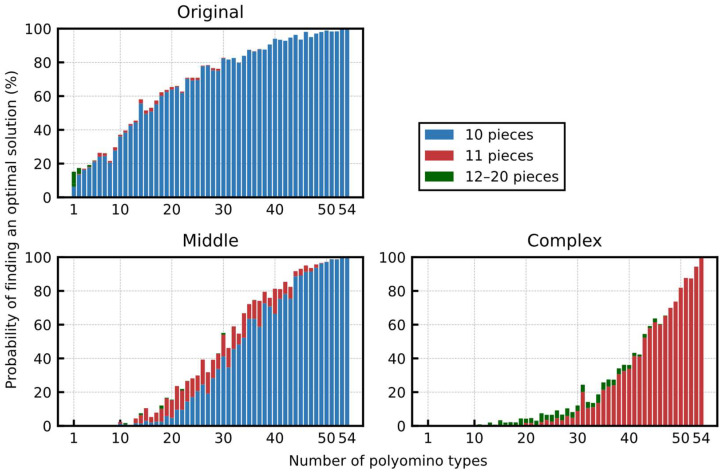
Probability of finding an optimal solution and the breakdown of the number of placed polyominoes for the number of types of polyominoes.

When all the 54 types of polyominoes were available, an optimal solution was found with a high probability and a small number of simulation steps. Therefore, to verify the performance when the number of available polyomino types becomes smaller, we performed experiments by randomly removing the polyominoes from the set of polyominoes of the basic 54 types. Figure 13 shows the probability of finding an optimal solution and the breakdown of the number of placed polyominoes for the number of types of polyominoes. Furthermore, $t_{max}$ is fixed at 500, which was considered large enough (for reference, the results for various $t_{max}$ are shown in Figure S1 in the Supplementary Material). Because the shape of the board became more complicated from Original to Complex, the probability of finding an optimal solution decreased more rapidly, when gradually removing the polyominoes from the set. For the Middle and Complex boards, it was almost impossible to obtain any optimal solution with randomly selected ten types of polyominoes from the original set. This is because a complex local part of a board tends to have few candidate polyominoes to cover it, and all such candidates may have been removed. In contrast, considering the Original board, it was possible to cover the board with fewer types of polyominoes. Even when we randomly selected only ten types of polyominoes, the optimal solution was found with a probability of approximately 40%.

## 6. Discussion

### 6.1. Optimization of the Coefficients of the Hamiltonian

For the optimization of the coefficients of the Hamiltonian function, we used Optuna [23], a Bayesian optimization library in Python. Bayesian optimization makes it possible to perform the parameter search efficiently, which is extremely time-consuming when grid search is used. Based on the preliminary experiments, the search range of each parameter was set as shown in Table 5, and the number of iterations in the Bayesian optimization was set to 1000 to find the optimal parameters. In each experiment, the obtained parameters were rounded to three digits.

Among the searched parameters, a positive correlation was found between coefficient B of the $H_{2}$ term, which restricts the overlap of polyominoes, and coefficient F of the $H_{6}$ term, which suppresses all the pairs of neurons. This is to maintain a balance between the $H_{2}$ term (which provides a positive bias value for each neuron) and the $H_{6}$ term (which provides a penalty for each pair of neurons equally). No significant correlation was observed among the other parameters.

### 6.2. Proposal of a New Term That Minimizes the Total Number of Polyominoes Placed

We consider the generalized polyomino puzzle with various sizes of polyominoes in Section 5, and the total number of placed polyominoes is minimized by the effect of the $H_{6}$ term, which restricts all the pairs of neurons. However, the $H_{6}$ term is a guiding term whose effect is ignored at the end of the simulation by parameter γ. Therefore, considering the current design of the Hamiltonian, the final value of the Hamiltonian is the same, regardless of the size of the polyominoes that cover the board. To explicitly differentiate the final Hamiltonian value depending on the size of the placed polyominoes, we designed an additional term that is inversely proportional to the size of the polyominoes as follows:

(14) $$H_{7}=\sum_{i=1}^{Q}\frac{V_{i}}{a_{i}}\;.$$

Adding Equation (14) to Equation (13), the Hamiltonian is modified as follows:

$$H=BH_{2}+CH_{3}+\gamma \left(DH_{4}+EH_{5}+FH_{6}\right)+GH_{7}\;.$$

The weights $W_{ij}$ between the neurons and biases $\theta_{i}$ are obtained as follows:

$$W_{ij}=\{\begin{array}{rr} -B\lambda_{ij}-Cf\left(i,j\right)+D\gamma g\left(i,j\right)-F\gamma & \mathrm{if}\;i\neq j\;,\\ 0 & \mathrm{otherwise}\;, \end{array}\;\theta_{i}=\frac{B}{2}a_{i}+E\gamma h_{i}-\frac{G}{a_{i}}\;.$$

We performed the same experiment as in Section 5.2.2 using the modified Hamiltonian. For the coefficients of the modified Hamiltonian, we used Bayesian optimization to determine the values shown in Table 6.

**Figure 14:**
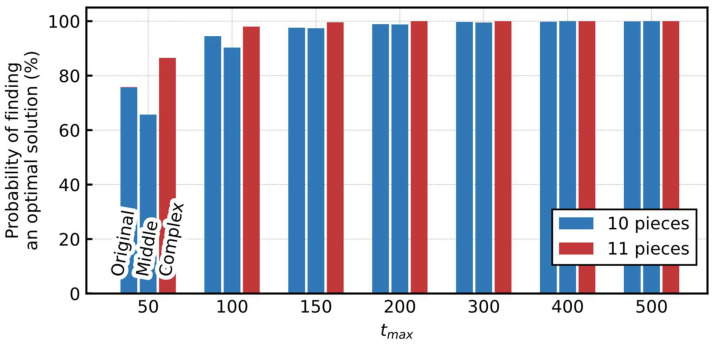
Probability of finding an optimal solution and the breakdown of the number of placed polyominoes for various $t_{max}$ with modified Hamiltonian.

Figure 14 shows the results of the experiment. Although the probability of finding an optimal solution at $t_{max}=50$ increases slightly, no significant improvement is observed overall. Moreover, there is no change in the total number of placed polyominoes, and the introduction of the $H_{7}$ term may assist in finding an optimal solution when the problem size increases.

### 6.3. Possibility of Application for Drug Discovery

Recently, fragment-based drug discovery (FBDD) has attracted much attention in the field of drug discovery. The method evaluates a drug candidate compound by the combination of fragments of the compound [15,24,25]. The number of unique fragments included is rather saturating, even when the size of the compound database grows very rapidly [24]. Considering this context, several methods have been proposed for solving the problems appearing in the FBDD as combinatorial optimization problems. For example, eHiTS performs a fragment-based docking simulation by formulating the problem as a weighted maximum clique-finding problem [25]. In addition, Banchi et al. solved a similar weighted maximum clique problem by emulating the Gaussian boson sampler (GBS), which is a type of optical quantum computer [15].

**Figure 15:**
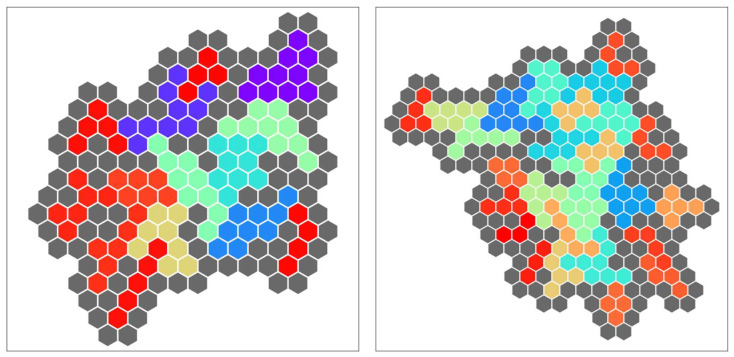
Examples of the problem with polyhexes solved in our software simulator.

The problem of filling the pocket of a target protein with fragments is similar to the generalized polyomino puzzle of covering a board with polyominoes. Therefore, we consider that the proposed Hamiltonian can be applied to drug discovery in the future. The fragments have varying sizes, and each fragment can be used zero or more times. Thus, our extension of the generalized polyomino puzzle illustrated in Section 5 is similar to the real-world problem. We needed a better resolution for representing the spherical shape of a protein pocket; therefore, we adopted the same Hamiltonian function with polyhex tiles (Figure 15).

**Figure 16:**
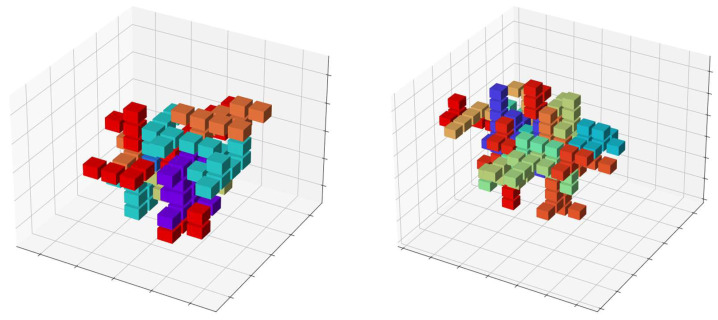
Examples of 3D problems with polycubes solved in our software simulator.

However, there are still huge differences between them. For example, regarding the FBDD, the space to be filled is 3D instead of 2D, and the pieces filling the space will take more complex shapes. Figure 16 shows examples of the Hamiltonian proposed in Section 4.2, applied to a problem with more complex pieces in 3D by using a cube instead of a square. We performed a series of computer experiments on these problems; however, the details are not presented in this paper.

One further difference is the necessity of satisfying constraints. For drug discovery, filling the 3D space, called the binding pocket, in the drug target protein is not the main aim, but rather designing more stable molecules in the binding pocket. This means that having some space remaining is acceptable. Furthermore, as the binding pocket has some flexibility, there is tolerance for overlapping pieces. This means that states that do not strictly satisfy the constraints may still be valuable. Considering this characteristic, some space on the board and overlapping of polycubes are allowed.

More importantly, we need to optimize the space-filling and the physicochemical interactions such as electrostatic interaction, hydrogen bonding, and hydrophobic interaction between the atoms. By defining a reward in each cube of the board (for each atom type), it is also possible to design the space-filling problem to maximize the sum of the rewards defined in each cube with soft constraints. When applying the Hamiltonian proposed in this study to this problem, we can introduce a reward $R_{n}$ for each cube n in the $H_{2}$ term as follows:

$$H_{2}=\frac{1}{2}\sum_{n=1}^{N}R_{n}{\left(\underset{i\in L_{n}}{\sum }V_{i}-1\right)}^{2}+\frac{1}{2}\sum_{n=1}^{N}R_{n}\sum_{i\in L_{n}}V_{i}\left(1-V_{i}\right)\;.$$

However, there are more difficult technical issues that need to be solved. First, the variety of possible fragments corresponding to the types of polycubes is enormous. For example, Yanagisawa et al. [24] demonstrated that the total number of types of fragments reached 263,319 for compounds registered in the ZINC12 database [26], a collection of commercially available chemicals. Therefore, instead of dealing with all these chemically observable fragments, it is necessary to select a set of core and important fragments that can be used to navigate the early stages of drug design. It is also necessary to consider the fine rotation of the fragments in the 3D space, and it may require a huge number of neurons or spins. Therefore, it is important to narrow down the number of candidates in advance, depending on the local information of the protein pocket space. Furthermore, although the polyomino puzzles only consider the contact between the polyominoes, the fragments on molecules have chemical bonds. In principle, these issues can be addressed by modifying the Ising model treated in this study; however, the number of spins will be too large unless it can be reduced using domain knowledge.

## 7. Conclusions

In this study, we considered a standard polyomino puzzle in which the board is covered with a given number of polyominoes, designed an improved Hamiltonian function, and proposed a method to obtain solutions efficiently using the Ising model. Moreover, we considered the generalized polyomino puzzle with relaxed restrictions on the number of times the polyominoes could be used, the various polyomino sizes, and the flexibility of the board shape. To verify the performance of the proposed method in finding the optimal solution, we conducted evaluation experiments on a software simulator of the Hopfield neural network. Using our formulation, we were able to solve the standard pentomino puzzle—with the problem represented by 1928 spins—in more than 90% of the cases, whereas the models proposed in previous studies had difficulty solving them. Furthermore, several generalized polyomino puzzles were solved approximately 100% of the time with a small number of simulation steps. In addition, we consider the possibility of future applications to real problems such as fragment-based drug discovery. When dealing with combinatorial optimization problems inherent in real-world problems, the scale of the model to be solved can be enormous if we apply a simple modeling approach that treats all candidates equally. Therefore, it is necessary to keep the size of the problem compact by considering the specific domain knowledge and local properties of the problem. A theoretical study aimed at improving the design method of the Ising model and efficiently controlling the dynamics of the system to handle larger-scale optimization problems should also be considered.

## Figures and Tables

**Table 1:** Optimized coefficients of the Hamiltonian for the pentomino puzzle.

Problem	A	B	C	D	E	F
Pentomino puzzle	4.29	2.16	8.38	0.246	0.0358	1.95

**Table 2:** Optimized coefficients of the Hamiltonian for the generalized polyomino puzzles.

Problem	B	C	D	E	F
Original	3.09	5.64	0.777	0.430	4.76
Middle	1.17	3.98	0.511	0.671	2.36
Complex	2.66	3.31	0.129	0.164	4.83

**Table 3:** Number of types of polyominoes for each size. (The polyominoes that were used in the experiments are shown in bold).

Name	Size of Polyomino	No. of Types
monomino	1	1
domino	2	1
**tromino**	**3**	**2**
**tetromino**	**4**	**5**
**pentomino**	**5**	**12**
**hexomino**	**6**	**35**
heptomino	7	108
octomino	8	369

**Table 4:** Optimized coefficients of the Hamiltonian for the generalized polyomino puzzle with various sizes of polyominoes.

Problem	B	C	D	E	F
Original	3.38	5.70	0.615	0.518	3.91
Middle	1.41	5.05	0.647	0.328	3.31
Complex	1.81	5.56	0.482	0.407	3.29

**Table 5:** Search ranges for the parameters.

Parameter	Range
A	[0,5]
B	[0,5]
C	[0,10]
D	[0,1]
E	[0,1]
F	[0,5]

**Table 6:** Optimized coefficients of the Hamiltonian with an additional $H_{7}$ term for the generalized polyomino puzzle with various sizes of polyominoes.

Problem	B	C	D	E	F	G
Original	3.21	5.86	0.640	0.489	3.61	2.59
Middle	1.33	4.97	0.606	0.344	2.96	2.67
Complex	2.16	5.68	0.469	0.384	3.34	2.37

## Data Availability

The implementation and experimental data are open-sourced at https://github.com/akiyamalab/NNSim (accessed on 17 February 2022) under the MIT license.

## Supplementary Materials

The following are available online at https://www.mdpi.com/article/10.3390/e24030354/s1, Figure S1: Probability of reaching the optimal solution for the number of polyomino types and $t_{max}$.
