# Supplementary material for: Solving Generalized Polyomino Puzzles Using the Ising Model
Source: Entropy (Basel). 2022 Feb 28;24(3):354. doi: 10.3390/e24030354 (PMC8947422; doi:10.3390/e24030354)
Supplement: Supplementary file 1 [file entropy-24-00354-s001.zip › entropy-1566393-supplementary.pdf]

Supplementary Materials

Solving Generalized Polyomino Puzzles Using the Ising Model

Kazuki Takabatake, Keisuke Yanagisawa, and Yutaka Akiyama*

Department of Computer Science, School of Computing, Tokyo Institute of Technology, Meguro-ku, Tokyo 152-8550, Japan

takabatake@bi.c.titech.ac.jp (K.T.); yanagisawa@c.titech.ac.jp (K.Y.).
* Correspondence: akiyama@c.titech.ac.jp

Figure S1 shows the probability of reaching the optimal solution for various number of polyomino types and t_{max}. For each of three boards, the probabilities of reaching the optimal solution at $t_{max} = 200$ and $t_{max} = 500$ were almost equal, indicating that t_{max} of approximately 200 was sufficient for the search performance. When the size of the polyomino set was small, there was no difference by t_{max} because the scale of the problem was small, while the difference by t_{max} increased when the size of the polyomino set was large.

In the case of Original board with shorter simulation time, $t_{max} = 50$, there was a saturation of probability improvement when the number of polyominoes exceeds 30, and even degrading when it was over 40. This may suggest two different aspects of increasing candidates: the richer polyomino set makes the problem easier, but the increasing number of total neurons makes it difficult to find minimums quickly.

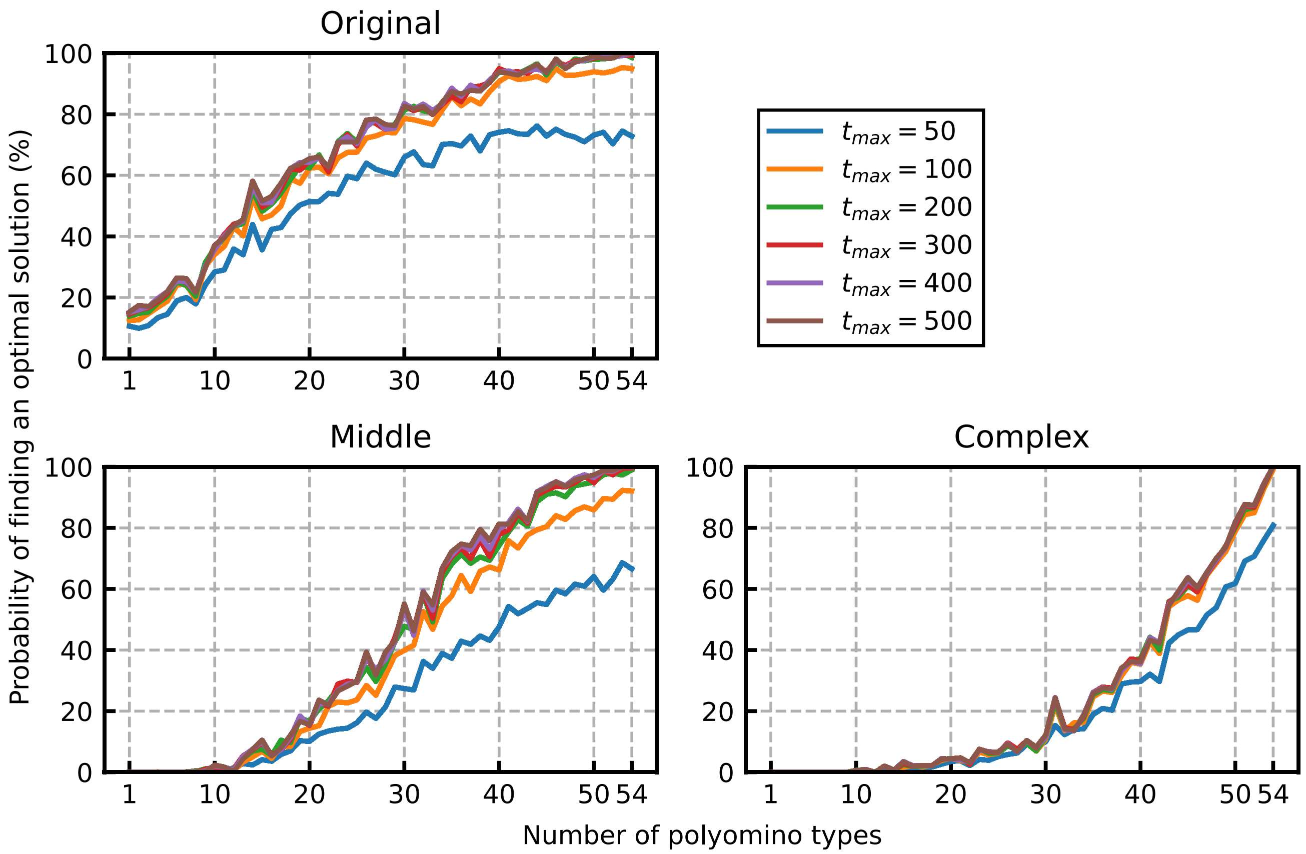

Figure S1. Probability of reaching the optimal solution for the number of polyomino types and t_{max}.
